# Discovering combinatorial interactions in survival data

**DOI:** 10.1093/bioinformatics/btt532

**Published:** 2013-09-13

**Authors:** David A. duVerle, Ichiro Takeuchi, Yuko Murakami-Tonami, Kenji Kadomatsu, Koji Tsuda

**Affiliations:** ^1^Computational Biology Research Center, National Institute of Advanced Industrial Science and Technology, Tokyo, Japan, ^2^Department of Computer Science, Nagoya Institute of Technology, Nagoya, Japan, ^3^Division of Molecular Oncology, Aichi Cancer Center, Nagoya, Japan and ^4^Department of Molecular Biology, Nagoya University Graduate School of Medicine, Nagoya, Japan

## Abstract

**Motivation:** Although several methods exist to relate high-dimensional gene expression data to various clinical phenotypes, finding combinations of features in such input remains a challenge, particularly when fitting complex statistical models such as those used for survival studies.

**Results:** Our proposed method builds on existing ‘regularization path-following’ techniques to produce regression models that can extract arbitrarily complex patterns of input features (such as gene combinations) from large-scale data that relate to a known clinical outcome. Through the use of the data’s structure and itemset mining techniques, we are able to avoid combinatorial complexity issues typically encountered with such methods, and our algorithm performs in similar orders of duration as single-variable versions. Applied to data from various clinical studies of cancer patient survival time, our method was able to produce a number of promising gene-interaction candidates whose tumour-related roles appear confirmed by literature.

**Availability:** An R implementation of the algorithm described in this article can be found at https://github.com/david-duverle/regularisation-path-following

**Contact:** dave.duverle@aist.go.jp

**Supplementary information:** Supplementary data are available at *Bioinformatics* online.

## 1 INTRODUCTION

From their inception, high-dimensional genomic data, such as obtained through genome-wide expression microarrays, have been used to identify genes that affects survival or tumour reoccurrence time spans among cancer patients (Bøvelstad *et al.*, 2007; Van De Vijver *et al.*, 2002). Survival data generally contain partially known observations (e.g. when clinical follow-up of the patient ends before a decisive event) requiring the use of regression models that can specifically handle censored data. Cox proportional hazards model (Cox, 1972) is one such model that combines advantages of both parametric and non-parametric approaches to statistical inference, making it ideally adapted to the type of data obtained in clinical trials.

Owing to the high dimensionality and small sample size of gene expression data, it is desirable to add a penalization component in fitting the Cox model (Dudoit *et al.*, 2002; Ghosh, 2003; Van De Vijver *et al.*, 2002), with 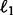-norm often preferred for its ability to drive sparsity of the model and select a concise set of variables (gene expression values, mutation types, etc.) (Gui and Li, 2005; Tibshirani *et al.*, 1997). Different methods have been suggested (Gui and Li, 2005; Lin and Wei, 1989; Park and Hastie, 2007) for fitting 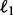-penalized Cox model. Park and Hastie (2007), in particular, proposed a method to compute the *regularization path* of 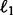-penalized Cox model, producing a series of Cox models that have different levels of complexity and sparsity.

As for many models in systems biology, it has been widely shown (Hanahan and Weinberg, 2000; Tibshirani *et al.*, 2002) that the gene regulatory pathways of cancer involve non-linear gene interactions. Although models based on linear combinations of gene expression may accurately approximate more complex interactions for some tasks, it can be desirable to specifically identify combinatorial covariates for such purpose as the identification of synthetic lethal genes (Kaelin, 2005). However, all current methods rely on the ability to enumerate potential input variables: although it is computationally feasible to examine each single gene in such a way (even for a large microarray), issues of exponential complexity quickly arise when considering interactions between more than one gene at a time.

In this article, we extend the approach in Park and Hastie (2007) to handle combinatorial interactions among genes. We deal with issues of combinatorial explosion and computational complexity by taking advantage of itemset mining techniques (Uno *et al.*, 2004). Using this approach, virtually limitless combinations of genes and phenotypes, grouped in itemsets of boolean variables, can be used as single predictor variables in the model. Our proposed algorithm computes the regularization path of 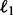-penalized Cox models that account for the effects of combinatorial gene interactions on survival.

Beyond proportional hazards models, our itemset-based method can be applied to any regression model with convex loss, each time making use of the input’s structure and sparsity to sidestep complexity issues, while at the same time guaranteeing that events along the regularization path (values of the regularization parameter for which a change occurs in the model structure) are exhaustively explored.

In the rest of this article, section 2 first outlines our general approach for adapting existing path regularization techniques to work with patterns of discretized input features instead of single continuous values. Section 3 details the mathematical basis for our algorithm and illustrates its application to proportional hazard models using Cox’s partial likelihood as loss function (with further detailed proofs as Supplementary Material). Finally, section 4 presents qualitative and quantitative results obtained by applying our method to different survival datasets.

## 2 APPROACH

### 2.1 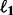-penalized maximum likelihood estimation

A common defining feature to many major regression models, such as generalized linear models (GLM) or previously mentioned Cox model, is the use of a loss function to fit the parameters of otherwise analytically intractable problems. Adding an 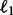 penalty term to the original loss criterion results in the typical estimation problem:
(1)
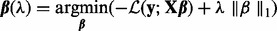

where 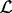 denotes the log-likelihood function with respect to the given data 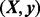, 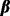 is the vector of coefficients that needs to be estimated and λ the regularization parameter.

For values of λ tending towards infinity, all coefficients in 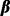 will be forced to 0, whereas as λ decreases, more coefficients will have non-null values (i.e more predictor variables will be used in the model estimation).

### 2.2 Regularization path-following algorithm

**Fig. 1.**
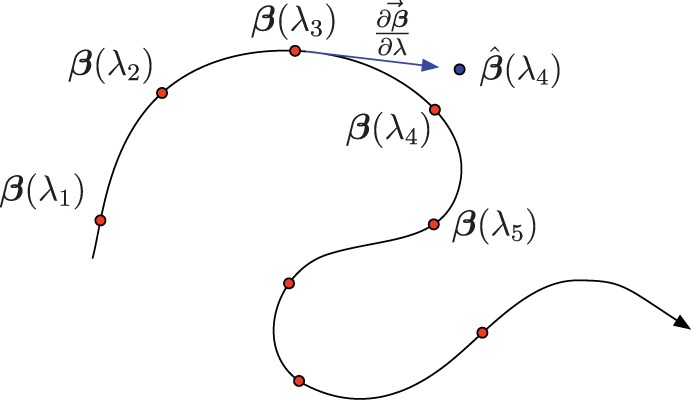
Schematic representation of the regularization path in the space of 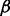. Successive values of 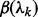 can be approximated using 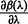

Among various methods for solving 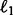-regularized problems similar to (1), the use of so-called ‘regularization path-following’ algorithms (Hastie *et al.*, 2005; Park and Hastie, 2007) is of particular interest for their ability to finely control the number of active variables in the model, regardless of the dimensionality of the input. The general idea behind path-following is to study variations of the λ parameter in the space of 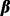 coefficient values (see Fig. 1): by decreasing the value of λ, starting from the maximum 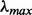 for which 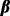 is non-null, we can find a sequence of all discrete values of λ, for which new coefficients of 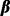 change between null and non-null (corresponding to a particular predictor variable exiting or entering the regression model). The resulting sequence of 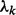 and associated optimal 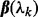 allow us to model the data at varying levels of sparsity.

Park and Hastie (2007) suggested a path-following algorithm for 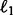-regularized GLM that uses a predictor-corrector approach to efficiently find all 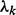 and the coefficients of the model associated with each level of regularization. If we define the ‘active set’, 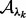, as the set of non-null indices in the coefficient vector 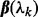, their algorithm can be defined as a loop over four main steps:

1. **Predict:** Starting with a known 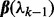 and 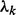: the next target value of λ, estimate 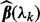 using a piecewise linear approximation of 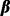, under the assumption that 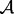 remains unchanged.
2. **Correct:** Solve the associated convex optimization problem to find the exact value of 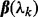 (using the linear approximation as a warm start).
3. **Update active set:** By confronting the new values of 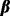 to the optimality conditions of the problem, update 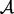 (i.e. add/remove predictors from the model). Repeat step 3 if necessary to adjust 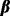.
4. **Decrement λ:** Analytically find the exact value of 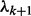, at which the active set will next change.

It is worth noting that, when an 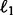-regularized model is fitted to high-dimensional small sample data, sparse models are usually selected (based on some model selection criteria). Therefore, we do not really have to compute the ‘entire’ regularization path (from 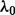 to 0). The algorithm is usually terminated for a value of λ where the size of the active set 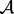 is still much smaller than the input dimension.

Because steps 1 and 2 only use variables in the current active set 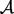, they can be performed at little computing cost for values of λ where 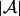 remains much smaller than the number of variables. Steps 3 and 4 require solving simple equations for each possible input variable (in linear time of the input’s dimension).

In their work, Park and Hastie (2007) showed that, along with GLM, their algorithm could also easily be applied to the Cox proportional hazards model. In fact, it can be shown that their results hold for any loss-based model fitting task, provided a loss function that exhibits certain mathematical properties (see section 3 and Supplementary Material).

### 2.3 Finding combinatorial covariates

When the linear model is extended to combinatorial interaction terms, the input dimension increases exponentially because of the combinatorial explosion of gene interactions. Of the steps enumerated in section 2.2, the *predictor* and *corrector* steps only deal with the small subset of covariates currently in the active set 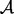, and therefore do not need to be changed. On the other hand, updating the active set in step 3 and finding the next value of λ at which an update event will occur in step 4, both potentially require examining a number of feature combinations that grows exponentially with the order of the interactions considered.

One practical approach to dealing with issues of combinatorial explosion and computational complexities in steps 3 and 4 is to take advantage of the input’s structure to efficiently explore its space. By discretizing our input (gene expressions or other clinical data) and considering all possible sets of such binary variables, we can use itemset mining techniques (Saigo *et al.*, 2007; Uno *et al.*, 2004) to preserve the computational efficiency of the path-following algorithm despite a high dimensional input.

We show that step 3 can be reduced to a weighted itemset mining problem, easily solvable using existing optimization techniques (see Methods section 3.1.3), whereas step 4 requires solving a particular form of fractional programming problem, for which we developed an efficient pruning approach (see Methods section 3.1.4). Our method can therefore overcome those computational complexity issues, and identify complex interactions (between two or more factors) that contribute to the response model, at varying degrees of sparsity (controlled by the penalization component).

### 2.4 Application to Cox proportional hazards model

We applied our modified version of the path-following algorithm to the Cox proportional hazards model, where patient survival (or any timed event) is used as a response, allowing for missing data because of right censorship. To estimate this model, we seek to maximize a so-called log partial likelihood function (see Methods section 3.2) for a given set of data. As predictors, we use discretized values of the gene expression levels (see section 4.1).

## 3 METHODS

In this section, we give a quick overview of the path-following algorithm first presented by Park and Hastie (2007) and the necessary changes to work on combinatorial interactions:

### 3.1 Path-following algorithm

Let 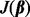 be the criterion from (1):
(2)
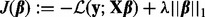



In the regularization path, we consider the optimal parameter vector 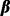 as a function of the regularization parameter λ, and represent the optimal parameter vector at λ as 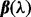. We can write the optimality condition as follows:
(3)
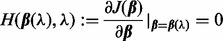



Our goal is to compute the path of solutions of (3) for all the λ. If we only consider the range of λ where the active set 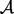 does not change (noting 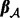: the restriction of 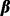 to the active set 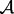), the partial change of the optimality condition (3) with respect to λ must satisfy:
(4)
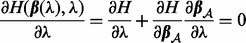



#### 3.1.1 Predictor step

In each predictor step, we assume that the current active set, 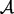, does not change. In the *k*-th predictor step, we use a linear approximation to predict 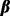 with the current active set:
(5)
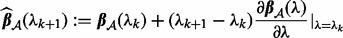



#### 3.1.2 Corrector step

We also assume that the active set 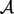 does not change during each corrector step. Any convex optimization algorithm can be used to minimize the penalized loss function (2). The use of 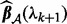 as an initial starting point ensures that an optimal solution can be found in a small number of iterations.

#### 3.1.3 Active set update

After each corrector step, it is necessary to identify all new features that should enter 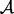. If we consider the set 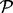 of all possible patterns, up to a given length, of binarized input features (e.g. ‘*gene A over-expressed and gene B under-expressed’*) and assign each such pattern an index value, for any 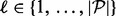, we note 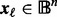 (where *n* is the total number of observations) the indicator vector for the matching pattern. Our goal is to identify such values of 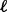 that contribute to minimize the loss function (2), and for which the matching value of the parameter vector 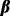 should be non-null (noted as 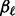 being ‘active’ and 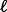 being in the ‘active set’ 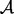).

With the feature notation 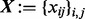, we define:
(6)
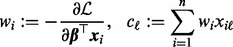

Assuming strong complementarity slackness, we obtain the following result (see Supplementary Material for detailed proof):

**Theorem 1**

(7)
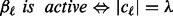

Therefore, if 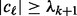 after the corrector step, 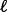 (and its associated parameter 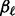) must be added to the active set 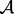.

If 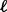 were an easily enumerable feature (such as in the case of single gene expression level), it would be computationally feasible to exhaustively enumerate all values of 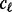 for all possible 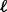. In our case, however, 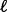 can match an arbitrarily long pattern drawn from the power set of all binarized features; the number of such features grows exponentially with the maximum size of the patterns, making the problem highly impractical for sets of >2 or 3 items. However, as long as 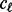 can be rewritten as linear sums of 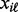, finding all such 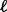 can be accomplished in reasonable time, using frequent itemset enumeration techniques.

Because the values *w_i_* in the linear sum defined in (6) do not depend on 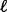 (and are constant for 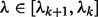), finding all items 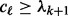 is equivalent to finding all itemsets with weighted support above 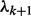 (the symmetrical problem of also finding 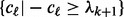 is then trivial). To solve this problem, we use the LCM program (http://research.nii.ac.jp/∼uno/codes.htm) (Uno *et al.*, 2004), which provides an exhaustive enumeration of frequent itemsets in guaranteed polynomial time per itemset.

If any variable is added to the active set 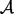, or removed (indices 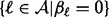), we go back to the corrector step (where the new values of 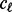 are first recomputed). These two steps are repeated until the active set does not change, thus guaranteeing that the solutions are optimal.

#### 3.1.4 Step length

To determine the optimal step length (the minimal value by which the regularization parameter must be decreased in order for the active set to change), we need to solve a similar problem, this time involving the ratio of two separate frequent itemset mining optimization problems.

If we define the step length:

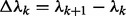

the minimum decrement of λ for which the active set 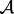 changes (a variable is added or removed), it can be shown (see Supplementary Material for detailed proof) that:

**Theorem 2**

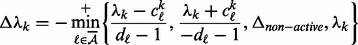

*where* 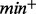 *is the smallest* strictly positive *value*, 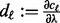 *and* 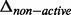 *are obtained by:*
(8)
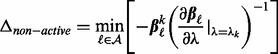

We note that 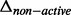 only depends on the variables in the active set and can be easily computed. On the other hand, much like in section 3.1.3, exhaustively computing the values of the first two expressions in (2) for all 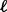 in 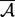 is not computationally feasible given the dimension of our input.

We designed an exploratory approach using bounds on each sub-problem to efficiently prune the search tree and drastically reduce the number of solutions explored.

First, we observe that both expressions can be rewritten as optimization problems of the form:
(9)
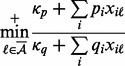

where 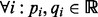 only depend on the variables in the active set 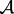 (and can therefore be easily computed) and 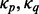: constant terms (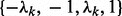).

We consider a relaxed form of (9), known as unconstrained fractional 0–1 programming, problem (Hammer *et al.*, 1968) and frequently encountered in the fields of scheduling or database query optimization (Hansen *et al.*, 1990):
(10)
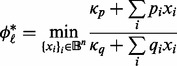

where *n* is the number of non-zero values for the itemset 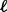 being considered. 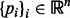 and 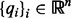.

Although the general form of this problem is shown to be NP-hard (by association to the well-known NP-complete *subset sum* decision problem), it has an easy polynomial solution (Boros and Hammer, 2002; Hammer *et al.*, 1968) if certain conditions hold. 

With the following notation, separating positive and negative terms in the sums of *p_i_* and *q_i_*:

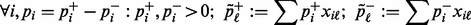


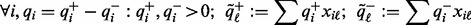

we have the following result:

**Theorem 3**

*For a given itemset* 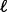
*, it is not necessary to explore any supersets of* 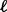 *if either of the following conditions holds:*

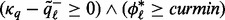


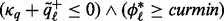

*where curmin is the current minimum value found by the algorithm up until itemset* 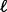
*.*

A much faster (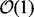), albeit slightly weaker, pruning condition can also be obtained (see proof in Supplementary Material):

**Theorem 4**

*For a given itemset* 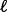
*, it is not necessary to explore any supersets of* 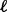 *if either of the following conditions holds:*

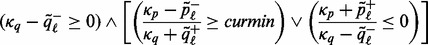


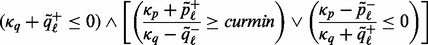

Although this pruning-based method loses some of its efficiency as the regularization parameter λ decreases and the model becomes less sparse, for the range of values of 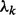 treated, it remains well within the reach of standard computing equipment (under a minute on a single 3.2 GHz CPU core).

### 3.2 Application to Cox proportional hazards model

To demonstrate the potential of our method, we applied it to the Cox model. This model uses survival data of the general form 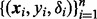, where 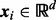 is the vector of risk factors, for instance gene expression levels. In practice the 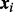 used by our method is vector of binary indicators of under- or over-expression (possibly in combination); 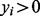 is the time observed (survival until an event or censoring); 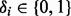 is a binary variable indicating whether an event has taken place (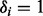) or the observation was right censored (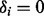).

The Cox regression model (Cox, 1972) for the hazard of death at time *t* can be expressed as:
(11)
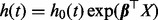

where 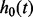 is the baseline hazard function, 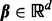 is the vector of parameters and 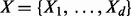 is the vector of risk factor variables with corresponding sample value of 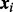 for the *i*-th sample.

However, it is not necessary to know 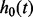 to infer the regression parameters, thanks to the use of the log partial likelihood function of the Cox model (Tibshirani *et al.*, 1997), defined as:
(12)
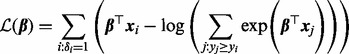



Refer to the Supplementary Material for the exact computation of the criterion 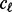 (6) in the case of the Cox proportional model.

### 3.3 Gathering synthetic candidates

To extract as many interaction candidates as possible, while avoiding the risk of overfitting the data, we repeatedly run the path-following algorithm on a randomly chosen subset of the input. It has been shown (Meinshausen and Bühlmann, 2010) that the use of such sampling method with regularized methods of variable selection provides a good estimator of the original data. On each run of the algorithm, we keep feature combinations that show a significantly improved predictive power over the linear models (likelihood ratio test *P*-value 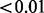). We aggregate all such combinations and rank them by Kaplan–Meier test *P*-value to produce a list of candidate interactions positively or negatively affecting the timed outcome.

As could be expected, a few combinations will tend to reoccur multiple times across successive iterations of the algorithm, whereas a large number only occurs once or twice. We hypothesized and verified *a posteriori* (see Supplementary Material) that combinations with low number of occurrences might be overfitting a particular iteration’s training subset and have poor generalization power. We therefore set an additional screening thresholds on the list of interactions, keeping only those that occur in at least four (out of 100) iterations. This threshold value was selected as giving the best compromise between ratio of false positives and overall number of interactions found (see details in Supplementary Material).

Independent testing shows remarkable stability of the list of selected interactions for a large-enough number of iterations. With our chosen occurrence and *P*-value thresholds, the final list of variables sees little change after ∼50 iterations (see plot in Supplementary Material). This trend is also confirmed when using an independent test: none of the rarely occurring combinations added in later iterations turn out to be significant in the test subset. For our experiment, we therefore set the total number of total iterations to 100, a value that once again seems to offer a good compromise between exhaustivity and the risk of false discovery.

## 4 EVALUATION

### 4.1 Datasets

To test our method, we used two datasets publicly available: survival studies of neuroblastoma (Oberthuer *et al.*, 2006) and breast cancer (Van De Vijver *et al.*, 2002) patients. In both studies, complementary DNA microarray assays of gene expression (10 163 probes for 9878 unique genes and 24 158 probes for 23 031 unique genes, respectively), along with (right-censored) survival data, were available for 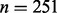 and 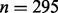 patients, respectively. In both cases, after setting aside a test subset (25% of all instances), the algorithm was iteratively applied on randomized subsets of the training data (95%) in a method similar to the leave-one-out procedure (Kearns and Ron, 1999).

For each study, gene expression data were normalized across arrays using standard methods (Yang and Thorne, 2003), then discretized in two binary classes depending on their distance to the mean (μ) using a threshold proportional to the standard deviation (σ): genes that are over-expressed (expression value above 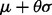, where θ is a thresholding parameter, set to 1.5 in this instance) or under-expressed (below 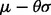).

To compare the higher-order interactions found by our method with a linear combination search, we ran the original Park and Hastie (2007) algorithm on the same training datasets and ranked the resulting variables found by the order in which they entered the regularized model. These ranks appear in the result tables under the column ‘single-variable rank’ (‘NA’, standing for ‘not applicable’, indicates a variable that did not appear in any of the models fitted by the single-variable version of the algorithm before one of its default termination conditions were reached). 

### 4.2 Analysis of breast cancer data

**Fig. 2.**
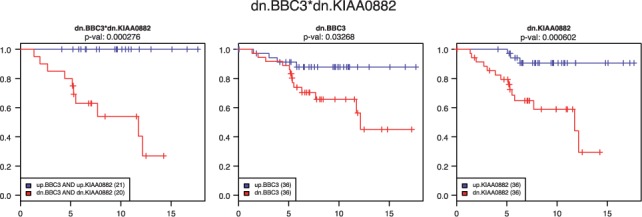
Kaplan–Meier plots for genes BBC3 and KIAA0882 (separately and in combination) in data used by Van De Vijver *et al.* (2002) (using test subset independent from training data used to compute Table 1)

The list of interactions found for Van De Vijver *et al.* (2002) (see Table 1) not only features a large number of genes strongly associated with breast cancer prognosis in the medical literature, such as SLC2A3 (Sternlicht *et al.*, 2006), CA9 (Span *et al.*, 2003), RAB6B (van’t Veer *et al.*, 2002), BBC3 (Cobleigh *et al.*, 2005) or KIAA0882 (Abba *et al.*, 2005), many of which do not appear at all in single-variable model fits (see single-variable ranks); it also features interesting examples of synthetic interactions: e.g. the Kaplan–Meier plot for the interaction between BBC3 and KIAA0882 (Fig. 2) shows perfect prediction of survival of all test samples (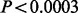), compared with the much less significant plot for BBC3 alone (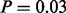), whereas a strong synthetic effect can be observed with BBC3 over-expressed (logrank *P*-value: 0.008, see plots in Supplementary Material).

**Table 1.** Interaction results for Van De Vijver *et al.* (2002)

Gene combination	LR test *P*-value	Logrank *P*-value	No. of occurrences	Test logrank *P*-value	Single-variable rank
up.SLC2A3 * up.CA9	**0.00153**	**0.000175**	**65**	**0.003432472**	**NA NA**
dn.Contig56307 * up.RAB6B	**0.00168**	**0.000392**	**15**	**0.09744396**	**NA NA**
dn.BBC3 * dn.KIAA0882	**5.21e-05**	**0.00043**	**22**	**0.0002761196**	**NA NA**
up.KIAA0964 * up.SLC2A3	0.000254	0.00132	23	0.04875811	NA NA
up.GADD153 * up.SLC31A1	0.0147	0.0022	5	0.2596054	540 NA
dn.Contig41887_RC * dn.KIAA0252	0.0151	0.00387	13	0.01261452	NA NA
up.RAD51C * up.TIMELESS	0.0367	0.0168	4	0.001651706	NA NA
up.TGFBI * up.ITGA5	0.0195	0.0298	11	0.2221538	NA NA
dn.Contig41887_RC * up.UGT8	0.000172	0.0329	51	0.003772726	NA NA

*Note*: Selected feature combinations, ranked by Kaplan–Meier *P*-value. Bonferroni-significant Kaplan–Meier test *P*-values are in bold (correction factor: m = 71). Total variables found with single-variable model: 585. Combinations of two genes (or more) are indicated by the symbol ‘*', while ‘up.' and ‘dn.' prefixes indicate up- and down-regulated genes, respectively.

Despite the overall small number of samples and difficulties to obtain good generalization power from such small training and test subsets, these results hold fairly well in test. Logrank *P*-values computed over an independent test subset for all selected combinations show 6 of 9 (66.7%) to be significant (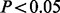), with 4 combinations (44%) still significant after Bonferroni correction for multiple-hypotheses testing.

### 4.3 Analysis of neuroblastoma data

The even smaller number of samples for Oberthuer *et al.* (2006) makes it difficult to obtain good generalized results (Table 2); however, the single interaction validated on the test subset (out of four interactions in total selected by our algorithm) not only shows strong predicting power on both subsets, but also involves two sequences strongly tied to breast cancer in literature. Locus BC046178 is associated with CENPW (previously known as C6orf173 or CUG2), a well-studied oncogene associated with apoptotic behaviours in tumour cells (Lee *et al.*, 2007, 2010). Probe Hs458148 is a match for multiple genes including RPL10: a ribosomal protein-coding gene that has been found to be over-expressed in breast cancer tumours (Nagai *et al.*, 2004). Although Hs458148 could also match other genes, its expression values in this dataset are highly correlated (Pearson’s coefficient: 0.63) with two other probes exclusively matching RPL10.

**Table 2.** Interaction results for Oberthuer *et al.* (2006)

Gene combination	LR test *P*-value	Logrank *P*-value	No. of occurrences	Test logrank *P*-value	Single-variable rank
up.BC046178 * up.Hs458148.20	**0.0131**	**2.16e-07**	**36**	**0.01003018**	**NA 67**
dn.THC1529413 * up.Hs172998.2	0.0199	0.00142	20	0.3228081	NA NA
dn.I_3233919 * up.USP1	0.0164	0.00561	61	0.2413684	NA 89
dn.U92981 * dn.SLC14A2	0.0147	0.0369	9	0.1264266	NA NA

*Note*: Selected feature combinations, ranked by Kaplan–Meier *P*-value. Bonferroni-significant Kaplan–Meier test *P*-values are in bold (m = 48). Total variables found with single-variable model: 474. Using same notations as Table 1.

### 4.4 Model validity and computation time

Although our goal is primarily not to create a predictor, but to gather input feature combinations (with promising synthetic lethality properties, in the case of cancer studies), we could still confirm that the model estimates produced by our method were sound and consistent with previous methods. Separating the original dataset in a training (75%), model-selection (12.5%) and test (12.5%) subsets and running nested cross-validation (100 iterations at the training level, each evaluated over 100 partitioning of the model-selection and evaluation subsets), we were able to compare the average log partial likelihood for both our algorithm and that of Park and Hastie (2007) (who use a 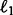-penalized path-following algorithm that only selects single variables, hereafter referred to as *single-variable algorithm* or *single-variable model*), both on the test subset.

Using the breast cancer survival data from Van De Vijver *et al.* (2002), our algorithm gave a mean log partial likelihood of −121.00 (SD: 27.56) compared with −117.10 (SD: 26.85) for the single-variable algorithm by Park and Hastie (2007), both significantly (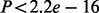) higher than the null model (−123.28, SD: 27.88), where no variables are used. With both algorithms, a large variance in the cross-validated results and overall middling performances are to be expected due to the small sizes of training, model-selection and testing subsets along with the typically high level of noise in microarray data. However, as the validation of the results in section 4.2 shows, there is still enough signal to detect meaningful covariates.

Additionally, we ran our algorithm on a randomized version of the breast cancer data, where survival data had been shuffled so as to no longer match its particular gene expression data. Using the same experimental set-up as described in 4.1, the algorithm produced only two significant interactions (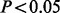): one of which only occurred once (and therefore would not be selected under normal conditions), whereas the other, with a *P*-value of 0.03, was no longer significant after Bonferroni correction (correction factor: 36) for multiple-hypotheses testing. This is to be contrasted with the multiple Bonferroni-significant interactions found in regular data (see section 4.2).

Computing time, although consistently longer for our algorithm was still within reasonable distance of the single-variable version: with similar termination conditions and the same input data, a single run of our path-following algorithm took on average <5 min (281 s ± 83 s) on a quad-core 3.2 GHz CPU, compared with a little under a minute for Park and Hastie (2007) (36 s ± 6 s).

## 5 CONCLUSION

In this article, we presented an algorithm to follow the regularization path of any 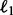-regularized linear model fitting, using combinatorial interactions as covariates. Although the path-following method has been applied to microarray data in the past (Park and Hastie, 2007), it was until now only able to deal with single-valued features, ignoring possible higher-order effect of gene interactions.

Our method makes uses of existing frequent itemset mining techniques and novel imports from fractional programming to avoid the intractability issues of combinatorial input and produce a regression model of accuracy and run time comparable with the linear case. By running multiple iterations of the algorithm on subsampled datasets, we can produce ordered lists of candidate interactions with strong predicting power.

The interactions found by applying our method to cancer study survival data include many genes that could not be found through linear models, yet show up in literature as strongly tied to these conditions, confirming the crucial importance of taking interaction effects into account to detect some of the weaker signal in gene expression data. Although most significant interactions found by our method on experimental data were limited to two or three genes, there are no theoretical limitations to the size of interactions that can be searched, at no particularly higher computational cost, setting this method apart from other recent work on penalized selection of interactions in high-dimensional data (Bien *et al.*, 2012).

The strong noise inherent to gene expression microarray likely prevents the detection of weaker signals between more than three genes, making it an attractive prospect to work with less noisy types of data where larger interactions might be detectable. In the future, we plan to extend our field of application to a wider range of biomedical data, such as the identification of SNP interactions (Schwender and Ickstadt, 2008), as well as leverage our model’s ability to deal with heterogeneous input, for example by including a wide range of clinical data in addition to the large-scale numeric data.

## Supplementary Material

Supplementary Data
