# Supplementary material for: Discovering combinatorial interactions in survival data
Source: Bioinformatics. 2013 Sep 13;29(23):3053–9. doi: 10.1093/bioinformatics/btt532 (PMC3834797; doi:10.1093/bioinformatics/btt532)
Supplement: Supplementary Data [file supp_29_23_3053__index.html]

Discovering combinatorial interactions in survival data — Supplementary Data 

# Discovering combinatorial interactions in survival data

## Supplementary Data

files

**Files in this Data Supplement:**

- Supplementary Data - pdf file
