# Supplementary material for: Discovering combinatorial interactions in survival data
Source: Bioinformatics. 2013 Sep 13;29(23):3053–9. doi: 10.1093/bioinformatics/btt532 (PMC3834797; doi:10.1093/bioinformatics/btt532)
Supplement: Supplementary Data [file supp_btt532_532supplementary_data_rev_3.pdf]

Discovering Combinatorial Interactions
Supplementary Material

August 19, 2013

1 Proof of Theorem 1: Optimality conditions

If we call $J(\boldsymbol{\beta})$ the criterion from the objective function:

$$J(\boldsymbol{\beta}) := -\mathcal{L}(\mathbf{y}; \mathbf{X}\boldsymbol{\beta}) + \lambda \|\boldsymbol{\beta}\|_1 \tag{1}$$

By writing the parameter vector $\boldsymbol{\beta} = \boldsymbol{\beta}^+ - \boldsymbol{\beta}^-$ with $\boldsymbol{\beta}^+ \in \mathbb{R}_+$ and $\boldsymbol{\beta}^- \in \mathbb{R}_+$, (1) can be reformulated as:

$$J(\boldsymbol{\beta}^+, \boldsymbol{\beta}^-) := -\mathcal{L}\left(\mathbf{y}; \mathbf{X}(\boldsymbol{\beta}^+ - \boldsymbol{\beta}^-)\right) + \lambda \sum_{\ell=1}^{d} (\beta_\ell^+ + \beta_\ell^-)$$

And the objective function as:

$$\min_{\boldsymbol{\beta}^+, \boldsymbol{\beta}^-} J(\boldsymbol{\beta}^+, \boldsymbol{\beta}^-) \tag{2}$$

$$\text{s.t.} \boldsymbol{\beta}^+ \geq \mathbf{0}, \boldsymbol{\beta}^- \geq \mathbf{0}. \tag{3}$$

To solve this constrained optimization problem, we define the Lagrangean:

$$L := J(\boldsymbol{\beta}^+, \boldsymbol{\beta}^-) - \sum_{\ell=1}^{d} \eta_\ell^+ \boldsymbol{\beta}_\ell^+ - \sum_{\ell=1}^{d} \eta_\ell^- \boldsymbol{\beta}_\ell^-, \tag{4}$$

where $\eta_\ell^+ \geq 0$ and $\eta_\ell^- \geq 0$, $\ell = 1, \ldots, d$, are the Lagrange multipliers.
At optimality, the following stationary conditions must be satisfied:

$$\frac{\partial L}{\partial \boldsymbol{\beta}_\ell^+} = -c_\ell + \lambda - \eta_\ell^+ = 0 \tag{5a}$$

$$\frac{\partial L}{\partial \boldsymbol{\beta}_\ell^-} = c_\ell + \lambda - \eta_\ell^- = 0 \tag{5b}$$

Where:

$$c_\ell := \frac{\partial \mathcal{L}(\mathbf{y}; \mathbf{X}\boldsymbol{\beta})}{\partial \boldsymbol{\beta}_\ell^+} = -\frac{\partial \mathcal{L}(\mathbf{y}; \mathbf{X}\boldsymbol{\beta})}{\partial \boldsymbol{\beta}_\ell^-} \tag{6}$$

for each $\ell = 1, \ldots, d$.
Since $\eta_\ell^+ \geq 0$ and $\eta_\ell^- \geq 0$ must be satisfied at the optimal solution, we can simplify the conditions as

$$-c_\ell + \lambda \geq 0, \tag{7a}$$
$$c_\ell + \lambda \geq 0, \tag{7b}$$

for $\ell = 1, \ldots, d$. In addition, complementary slackness conditions require:

$$\boldsymbol{\beta}_\ell^+ = 0 \quad \text{or} \quad \eta_\ell^+ = 0 \quad (\Leftrightarrow -c_\ell + \lambda = 0) \tag{8}$$

and

$$\boldsymbol{\beta}_\ell^- = 0 \quad \text{or} \quad \eta_\ell^- = 0 \quad (\Leftrightarrow \quad c_\ell + \lambda = 0). \tag{9}$$

Which eventually gives us:

$$\boldsymbol{\beta}_\ell \text{ is active} \implies |c_\ell| = \lambda. \tag{10a}$$
$$|c_\ell| < \lambda \implies \boldsymbol{\beta}_\ell \text{ is non-active}, \tag{10b}$$

Although the converse of the above relations are not necessarily true.
When strong complementary conditions are satisfied, we can extend the relations and obtain the following theorem:

Theorem 1.

$$\boldsymbol{\beta}_\ell \textit{ is active} \iff |c_\ell| = \lambda \tag{11}$$

2 Proof of Theorem 2: computation of step length

Defined as:

$$\Delta\lambda_k = \lambda_{k+1} - \lambda_k$$

the step length is the minimum decrement of λ for which the active set $\mathcal{A}$ changes (a variable is added or removed). Note that $\Delta\lambda_k < 0$ because we decrease λ in each step.
We need to consider two cases: (i) one of the non-active variable becomes active, (ii) one of the active variable becomes non-active.

Which non-active variable first enters the active set. To predict the largest $\Delta\lambda^k < 0$ at which one of the non-active variables enters to the active set $\mathcal{A}$, we use a linear approximation of c_ℓ:

$$\hat{c}_\ell^{k+1} := c_\ell^k + \Delta\lambda_k \frac{\partial c_\ell}{\partial \lambda}\Big|_{c_\ell = c_\ell^k} \tag{12}$$

where c_ℓ^k denotes the value of c_ℓ at λ_k.

From the optimality conditions in theorem 1, in order for $\boldsymbol{\beta}_\ell$ to be active, we must have:

$$|\hat{c}_\ell^{k+1}| = \lambda_{k+1} = \lambda_k + \Delta\lambda_k \tag{13}$$

Therefore, if we define:

$$d_\ell := \frac{\partial c_\ell}{\partial \lambda}\Big|_{c_\ell = c_\ell^k} \tag{14}$$

$\Delta\lambda_k$ is either:

$$\frac{\lambda_k - c_\ell^k}{d_\ell - 1} \quad \text{or} \quad \frac{\lambda_k + c_\ell^k}{-d_\ell - 1} \tag{15}$$

First active variable leaving the active set. To predict which active variable first becomes non-active, we use a linear approximation of $\boldsymbol{\beta}_\ell$. Let $\boldsymbol{\beta}_\ell^k$ be the $\boldsymbol{\beta}_\ell$ at λ_k. Then, we can predict the $\boldsymbol{\beta}_\ell$ at λ_{k+1} as follow:

$$\hat{\boldsymbol{\beta}}_\ell^{k+1} = \boldsymbol{\beta}_\ell^k + \Delta\lambda_k \frac{\partial \boldsymbol{\beta}_\ell}{\partial \lambda}\Big|_{\lambda = \lambda_k} \tag{16}$$

From the optimality conditions (theorem 1), $\boldsymbol{\beta}_\ell$ becomes non-active when the above expression is equal to 0. That is, in order for the first $\boldsymbol{\beta}_\ell$ to become non-active, the step length must be:

$$\Delta_{non-active} = \min_{\ell \in \mathcal{A}} \left[-\boldsymbol{\beta}_\ell^k \left(\frac{\partial \boldsymbol{\beta}_\ell}{\partial \lambda}\Big|_{\lambda=\lambda_k} \right)^{-1} \right] \tag{17}$$

Additionally, for $\Delta_k = \lambda_k$, the algorithm terminates as it leads to $\lambda^{k+1} = 0$. By combining (15) and (17), the step length is determined as:

$$\Delta\lambda_k = -\min_{\ell \in \bar{\mathcal{A}}}^{+} \left\{ \frac{\lambda_k - c_\ell^k}{d_\ell - 1}, \frac{\lambda_k + c_\ell^k}{-d_\ell - 1}, \Delta_{non-active}, \lambda_k \right\} \tag{18}$$

Rewriting d_ℓ as a linear sum. We have:

$$d_\ell := \frac{\partial c_\ell}{\partial \lambda} = \frac{\partial}{\partial \lambda}\left(\sum_{i=1}^n w_i x_{i\ell} \right) = \sum_{j=1}^n \frac{\partial}{\partial \theta_j} \left(\sum_{i=1}^n \frac{\partial C}{\partial \beta_{\mathcal{A}}} x_{i\ell} \right) \left(\frac{\partial \theta_j}{\partial \beta_{\mathcal{A}}} \right)^\top \left(\frac{\partial^2 C}{\partial \beta_{\mathcal{A}} \partial \beta_{\mathcal{A}}^\top} \right)^{-1} \frac{\partial \beta_{\mathcal{A}}}{\partial \lambda} \tag{19}$$

If we define:

$$v_i := \sum_{j=1}^n \left(x_{j\mathcal{A}} \left(\frac{\partial^2 C}{\partial \beta_{\mathcal{A}} \partial \beta_{\mathcal{A}}^\top} \right)^{-1} sgn(\beta_{\mathcal{A}}) \right) \frac{\partial^2 C}{\partial \theta_i \partial \theta_j} \tag{20}$$

We can write d_ℓ as a linear combination:

$$d_\ell = \sum_{i=1}^{n} v_i x_{i\ell} \tag{21}$$

where the coefficients v_i only depend on the variables in the active set ($\mathcal{A}$) and can therefore be easily computed.
By combining the above results, we obtain:

Theorem 2.

$$\Delta\lambda_k = -\overset{+}{\min_{\ell\in\bar{\mathcal{A}}}}\Big\{\frac{\lambda_k - c_\ell^k}{d_\ell - 1}, \frac{\lambda_k + c_\ell^k}{-d_\ell - 1}, \Delta_{non-active}, \lambda_k\Big\} \tag{22}$$

3 Proof for Theorem 3

To solve:

$$\overset{+}{\min_{\ell\in\bar{\mathcal{A}}}} \frac{\kappa_p + \sum p_i x_{i\ell}}{\kappa_q + \sum q_i x_{i\ell}} \tag{23}$$

We consider the relaxed form of problem 23:

$$\phi_\ell^* = \min_{\{x_i\}_i \in \mathbb{B}^n} \frac{\kappa_p + \sum_i p_i x_i}{\kappa_q + \sum_i q_i x_i} \tag{24}$$

in the specific case where the following condition holds:

$$\kappa_q + \sum_i q_i x_i > 0, \forall \{x_i\}_i \in \mathbb{B}^n \tag{25}$$

In that case, a partial optimal assignment y of $\{x_i\}_i$ can immediately be defined by:

$$y_i = \begin{cases} 1 \text{ if } (p_j < 0 \wedge q_j \geq 0) \vee (p_j = 0 \wedge q_j > 0) \\ 0 \text{ if } (p_j > 0 \wedge q_j \leq 0) \vee (p_j = 0 \wedge q_j < 0) \end{cases} \tag{26}$$

An exact solution ϕ_ℓ^* can then be found in $\mathcal{O}(n' \log n')$ (where n' is the number of variables not covered by y) using the method described in [1].
The method can be trivially extended to the case where the expression in condition (25) is strictly negative instead of positive and we have the following theorem:

Theorem 3. *For a given itemset ℓ, it is not necessary to explore any supersets of ℓ if either of the following conditions holds:*

$$\left(\forall \{x_i\}_i \in \mathbb{B}^n, \kappa_q + \sum_i q_i x_i > 0\right) \wedge (\phi_\ell^* \geq curmin)$$

$$\left(\forall \{x_i\}_i \in \mathbb{B}^n, \kappa_q + \sum_i q_i x_i > 0\right) \wedge (\phi_\ell^* \geq curmin)$$

4 Proof for Theorem 4

We consider problems of the form shown in 23:
With the following notations:

$$\forall i, p_i = p_i^+ - p_i^- : p_i^+, p_i^- > 0 \tag{27}$$
$$\forall i, q_i = q_i^+ - q_i^- : q_i^-, q_i^- > 0 \tag{28}$$

problem (23) then becomes:

$$\overset{+}{\min_{\ell \in \bar{\mathcal{A}}}} \frac{\kappa_p + \sum p_i^+ x_{i\ell} - \sum p_i^- x_{i\ell}}{\kappa_q + \sum q_i^+ x_{i\ell} - \sum q_i^- x_{i\ell}} \tag{29}$$

with $\forall i : p_i^+, p_i^-, q_i^+, q_i^- \geq 0$.
Rewritten, for concision (with $\tilde{p}_\ell^+ := \sum p_i^+ x_{i\ell}$, $\tilde{p}_\ell^- := \sum p_i^- x_{i\ell}$ etc.):

$$\overset{+}{\min_{\ell \in \bar{\mathcal{A}}}} \frac{\kappa_p + \tilde{p}_\ell^+ - \tilde{p}_\ell^-}{\kappa_q + \tilde{q}_\ell^+ - \tilde{q}_\ell^-} \tag{30}$$

In this formulation, $\tilde{p}_\ell^+$, $\tilde{p}_\ell^-$, $\tilde{q}_\ell^+$ and $\tilde{q}_\ell^-$ can be seen as the support of itemset ℓ (in our case, ℓ is a set of gene expression features of arbitrary size), weighted respectively by $\{p_i^+\}_i, \{p_i^-\}_i, \{q_i^+\}_i, \{q_i^-\}_i$.
We note that, for any superset ℓ' such that $\ell \subset \ell'$ (and therefore $supp(\ell') \leq supp(\ell)$), we have:

$$\tilde{p}_{min}^+ - \tilde{p}_\ell^- \leq \tilde{p}_{\ell'}^+ - \tilde{p}_{\ell'}^- \leq \tilde{p}_\ell^+ - \tilde{p}_{min}^- \tag{31}$$
$$\tilde{q}_{min}^+ - \tilde{q}_\ell^- \leq \tilde{q}_{\ell'}^+ - \tilde{q}_{\ell'}^- \leq \tilde{q}_\ell^+ - \tilde{q}_{min}^- \tag{32}$$

where x_{min} is a lower bound of x.
Due to the constraints in (29) ($\tilde{p}_\ell^+$, $\tilde{p}_\ell^-$, $\tilde{q}_\ell^+$ and $\tilde{q}_\ell^-$ are sums of positive values), we can use the lower bound 0 for $\tilde{p}_{min}^+, \tilde{p}_{min}^-, \tilde{q}_{min}^+$ and $\tilde{q}_{min}^-$.
From (31), we extract the following inequalities:

$$\kappa_q - \tilde{q}_\ell^- \geq 0 \implies \frac{\kappa_p - \tilde{p}_\ell^-}{\kappa_q + \tilde{q}_\ell^+} \leq \frac{\kappa_p + \tilde{p}_{\ell'}^+ - \tilde{p}_{\ell'}^-}{\kappa_q + \tilde{q}_{\ell'}^+ - \tilde{q}_{\ell'}^-} \leq \frac{\kappa_p + \tilde{p}_\ell^+}{\kappa_q - \tilde{q}_\ell^-} \tag{33}$$
$$\kappa_q + \tilde{q}_\ell^+ \leq 0 \implies \frac{\kappa_p + \tilde{p}_\ell^+}{\kappa_q - \tilde{q}_\ell^-} \leq \frac{\kappa_p + \tilde{p}^- + \ell' - \tilde{p}_{\ell'}^-}{\kappa_q + \tilde{q}_{\ell'}^+ - \tilde{q}_{\ell'}^-} \leq \frac{\kappa_p - \tilde{p}_\ell^-}{\kappa_q + \tilde{q}_\ell^+} \tag{34}$$

From these inequalities, we can draw theorem 4.

Theorem 4. *For a given itemset ℓ, it is not necessary to explore any supersets of ℓ if either of the following conditions holds:*

$$(\kappa_q - \tilde{q}_\ell^- \geq 0) \wedge \left[\left(\frac{\kappa_p - \tilde{p}_\ell^-}{\kappa_q + \tilde{q}_\ell^+} \geq curmin \right) \vee \left(\frac{\kappa_p + \tilde{p}_\ell^+}{\kappa_q - \tilde{q}_\ell^-} \leq 0 \right) \right]$$
$$(\kappa_q + \tilde{q}_\ell^+ \leq 0) \wedge \left[\left(\frac{\kappa_p + \tilde{p}_\ell^+}{\kappa_q - \tilde{q}_\ell^-} \geq curmin \right) \vee \left(\frac{\kappa_p - \tilde{p}_\ell^-}{\kappa_q + \tilde{q}_\ell^+} \leq 0 \right) \right]$$

where curmin is the current minimum value found by the algorithm up until itemset ℓ.

5 Computation of optimality conditions for Cox PH model

$$\mathcal{PL}(\boldsymbol{\beta}) \equiv \prod_{i:\delta_i=1} \frac{\exp(\boldsymbol{\beta}^\top \boldsymbol{x}_i)}{\sum_{j:y_j \geq y_i} \exp(\boldsymbol{\beta}^\top \boldsymbol{x}_j)} \tag{35}$$

Giving us the log-likelihood function:

$$\mathcal{L}(\boldsymbol{\beta}) = \sum_{i:\delta_i=1} \Big(\boldsymbol{\beta}^\top \boldsymbol{x}_i - \log\big(\sum_{j:y_j \geq y_i} \exp(\boldsymbol{\beta}^\top \boldsymbol{x}_j)\big)\Big) \tag{36}$$

And the criterion c_ℓ (6) for the optimality conditions can be written as:

$$c_\ell := \sum_{i:\delta_i=1} \big(x_{i\ell} - \sum_{j:y_j \geq y_i} w_{ij} x_{j\ell}\big) \tag{37}$$

for each $\ell = 1, \ldots, d$.
where:

$$w_{ij} := \frac{\exp(\boldsymbol{\beta}^\top \boldsymbol{x}_j)}{\sum_{y_{i'} \geq y_i} \exp(\boldsymbol{\beta}^\top \boldsymbol{x}_{i'})} \tag{38}$$

for $i, j = 1, \ldots, n$.
In the Cox PH model, $\frac{\partial H}{\partial \boldsymbol{\beta}_\mathcal{A}}$ can be written as:

$$\begin{aligned} \frac{\partial H}{\partial \boldsymbol{\beta}_\mathcal{A}} \quad &= \sum_{i:\delta_i=1} \Big\{ \sum_{j:y_j \geq y_i} w_{ij} \boldsymbol{x}_{j\mathcal{A}} \boldsymbol{x}_{j\mathcal{A}}^\top \\ &-\big(\sum_{j:y_j \geq y_i} w_{ij} \boldsymbol{x}_{j\mathcal{A}}\big)\big(\sum_{j:y_j \geq y_i} w_{ij} \boldsymbol{x}_{j\mathcal{A}}^\top\big)\Big\} \end{aligned} \tag{39}$$

where $\boldsymbol{x}_{j\mathcal{A}}$ is a vector of length $|\mathcal{A}|$ that only contains $x_{j\ell}, \ell \in \mathcal{A}$. This partial derivative can also be formulated as

$$\frac{\partial H}{\partial \boldsymbol{\beta}_\mathcal{A}} = X_\mathcal{A}^\top V X_\mathcal{A}, \tag{40}$$

where $X_\mathcal{A}$ is an $n \times |\mathcal{A}|$ matrix that contains $\boldsymbol{x}_{j\mathcal{A}}$, $j = 1, \ldots, n$, in each row, and V is an $n \times n$ matrix whose diagonal element is defined as

$$V_{jj} := \sum_{i:\delta_i=1} \big((w_{ij} - w_{ij}^2) I(y_j \geq y_i)\big), \ \ j = 1, \ldots, n. \tag{41}$$

and off-diagonal elements are defined as

$$V_{jj'} := \sum_{i:\delta_i=1} \big(-w_{ij} I(y_j \geq y_i) - w_{ij'} I(y_{j'} \geq y_i)\big),$$

$$j, j' = 1, \dots, n, \;\; j \neq j' \tag{42}$$

where $I(z)$ is the indicator function.
Combining them, we can write

$$\frac{\partial \boldsymbol{\beta}_{\mathcal{A}}}{\partial \lambda} = -(X_{\mathcal{A}}^{\top} V X_{\mathcal{A}})^{-1} \mathrm{sgn}(\boldsymbol{\beta}_{\mathcal{A}}). \tag{43}$$

From here on, we define:

$$\boldsymbol{e}_{\mathcal{A}} := (X_{\mathcal{A}}^{\top} V X_{\mathcal{A}})^{-1} \mathrm{sgn}(\boldsymbol{\beta}_{\mathcal{A}}). \tag{44}$$

The linear approximation for $\boldsymbol{\beta}$ can be computed as

$$\hat{\boldsymbol{\beta}}_{\mathcal{A}}(\lambda_{k+1}) \quad = \quad \boldsymbol{\beta}_{\mathcal{A}}(\lambda_k) - (\lambda_{k+1} - \lambda_k)\boldsymbol{e}_{\mathcal{A}}. \tag{45}$$

For all $\ell \in \bar{\mathcal{A}}$, $\boldsymbol{\beta}_\ell = 0$, therefore all w_{ij} in (38) can be computed using only the active parameters $\boldsymbol{\beta}_{\mathcal{A}}$ and (after reordering all $\boldsymbol{x}_i$ indices according to the values of y_i and δ_i), we can re-write (6) as a linear sum of the form $\sum_i \alpha_i x_{i\ell}$, with:

$$\alpha_i = 1 - \sum_{j \leq i} w_{ij} \tag{46}$$

d_ℓ can be computed as:

$$\frac{\partial c_\ell}{\partial \lambda} \tag{47}$$

with:

$$\frac{\partial c_\ell}{\partial \boldsymbol{\beta}_{\mathcal{A}}} = -\boldsymbol{x}_{:,\ell}^{\top} V X_{\mathcal{A}} \tag{48}$$

where $\boldsymbol{x}_{:,\ell} := [x_{1\ell}, \dots, x_{n\ell}]^{\top}$

$$\Delta\lambda^k = -\boldsymbol{\beta}_\ell^k \left(\frac{\partial \boldsymbol{\beta}_\ell}{\partial \lambda}\Big|_{\lambda=\lambda^k} \right)^{-1} \quad = \quad -\boldsymbol{\beta}_\ell^k / e_\ell \tag{49}$$

6 Stability of Sub-sampling Method

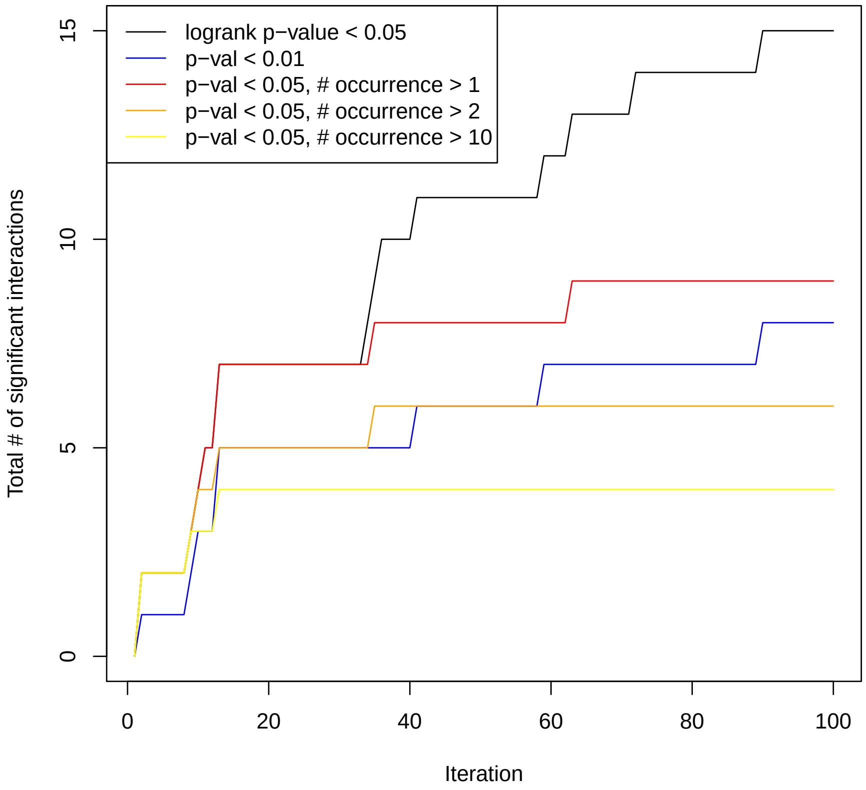

Figure 1: Number of new significant combinations (as selected by logrank test p-value) found on each iteration on the algorithm (using the same data as section 4.3). When considering only combinations that appear more than once, the algorithm shows remarkable stability after about 50 iterations (all new combinations are likely the result of overfitting).

7 Impact of occurrence count on significance

Number of occurrences	Interactions found	Significant on test set	Ratio
1 or more	31	14 (8)	0.45 (0.26)
2 or more	15	7 (5)	0.47 (0.33)
5 or more	7	4 (3)	0.71 (0.42)
10 or more	6	4 (3)	0.67 (0.50)

Table 1: Number of selected combinations significant (for significance level 0.05), as tested on independent test set. Values between parentheses indicate Bonferroni-corrected significance.)

8 Synthetic Effect of BBC3 and KIAA0882

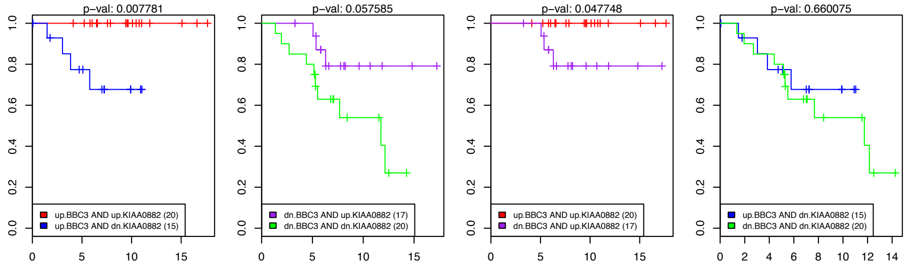

Figure 2: Kaplan-Meier plots within sub-groups of BBC3 and KIAA0882 show a clear synthetic effect of KIAA0882 when BBC3 is over-expressed.

References

[1] E. Boros and P.L. Hammer. Pseudo-boolean optimization. *Discrete applied mathematics*, 123(1):155–225, 2002.
